# Supplementary material for: Nanohybrid Assemblies of Porphyrin and Au10 Cluster Nanoparticles
Source: Nanomaterials (Basel). 2019 Jul 18;9(7):1026. doi: 10.3390/nano9071026 (PMC6669571; doi:10.3390/nano9071026)
Supplement: Supplementary file 1 [file nanomaterials-09-01026-s001.pdf]

Electronic Supporting Information: Nanohybrid Assemblies of Porphyrin and Au10 Cluster Nanoparticles

Mariachiara Trapani, Maria Angela Castriciano, Andrea Romeo, Giovanna De Luca, Nelson Machado, Barry D. Howes, Giulietta Smulevich, Luigi Monsù Scolaro

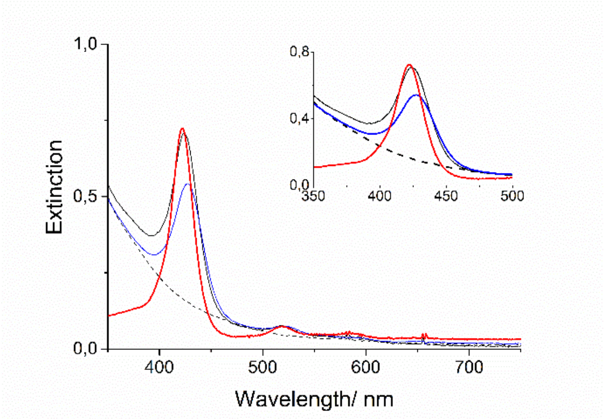

Figure S1. UV-vis spectra of a solution of Au10 (dotted line), immediately upon addition of tetra N- methylpyridinium porphyrin (H_2TMPyP^{4+}) (black line) and after 24 h (blue line). A bathochromic shift of the Soret band is observed compared to that of the free monomer (422 nm, red line). The inset shows an expanded view of the Soret region. Experimental conditions:[Au_{10}]= 125μM, [H_2TMPyP^{4+}]= 3μM.

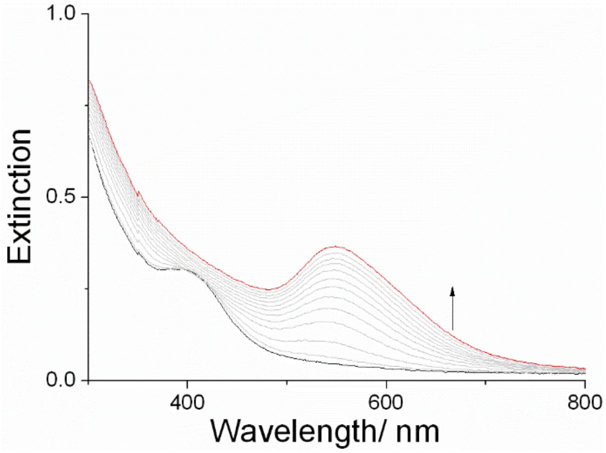

Figure S2. Spectral changes of Au10 clusters upon addition of HCl (pH 2) within 1 h. The first and the final spectra are shown as black and red lines, respectively. Experimental conditions: [Au_{10}] = 125 μM.

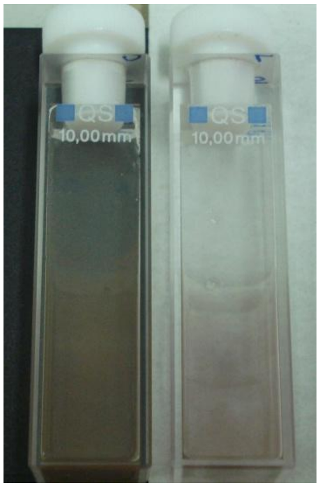

Figure S3. Photographs on dark and white backgrounds of quartz cells with a gold plating obtained from Au_{10} clusters solution at acidic pH after 24 h.

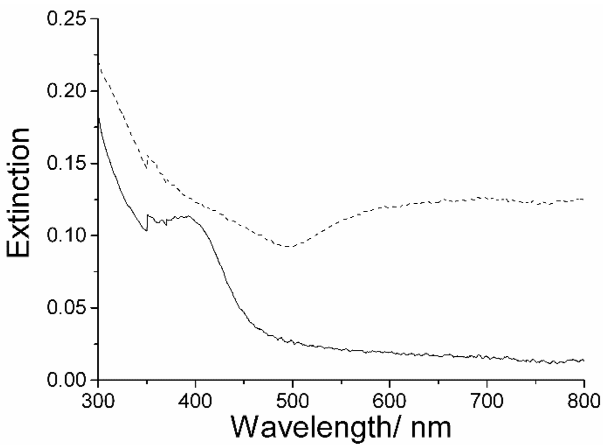

Figure S4. Extinction spectra of a solution of Au_{10}immediately (solid line) and after 1 hour from HCl addition (dotted line). Experimental conditions: [Au_{10}]= 30 μM, [Histidine]= 28 mM, pH 2.0.

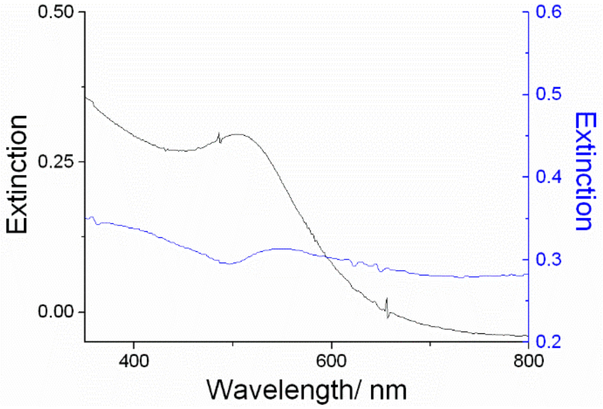

Figure S5. Extinction spectra of Au nanoparticles (NPs) in solution synthesized from histidine and $NaBH_4$ (black line) and upon HCl addition (pH 2) after 24 h (blue line). No deposit was observed on the cuvette walls after removing the solution.

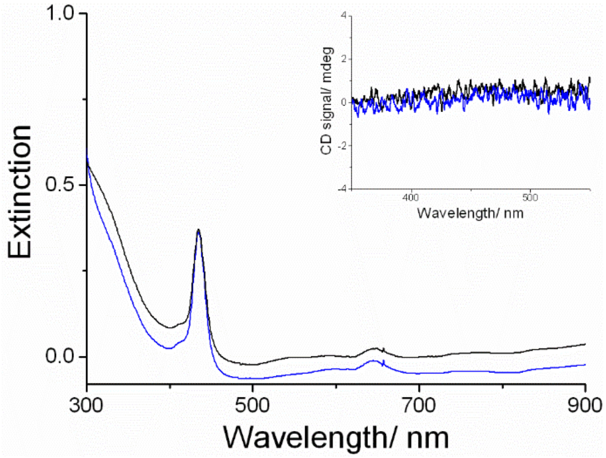

Figure S6. UV-vis and CD (inset) spectra of solutions of TPPS with Au_{10} clusters synthesized with D- and L- histidine (black and blue lines, respectively) at acidic pH, transferred after 24 h into a new cuvette. ([Au_{10}] = 125 μM, [TPPS] = 5 μM, pH 2.0).

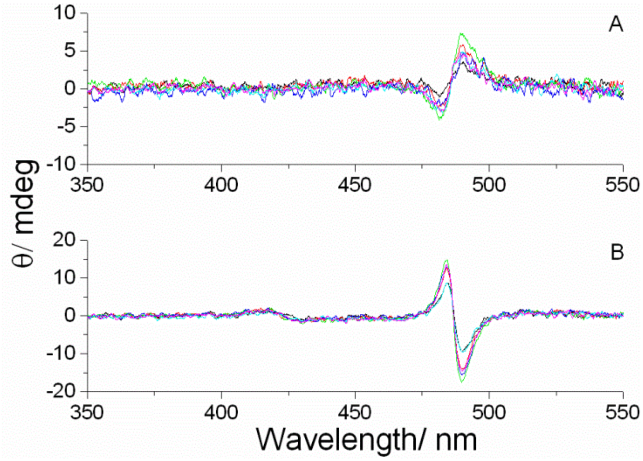

Figure S7. Circular Dichroism (CD) spectra of co-deposit of J aggregates and AuNPssynthesized by D- (**a**) and L-histidine (**b**). Spectra taken rotating the cuvettes at different angles with respect to the incident light.

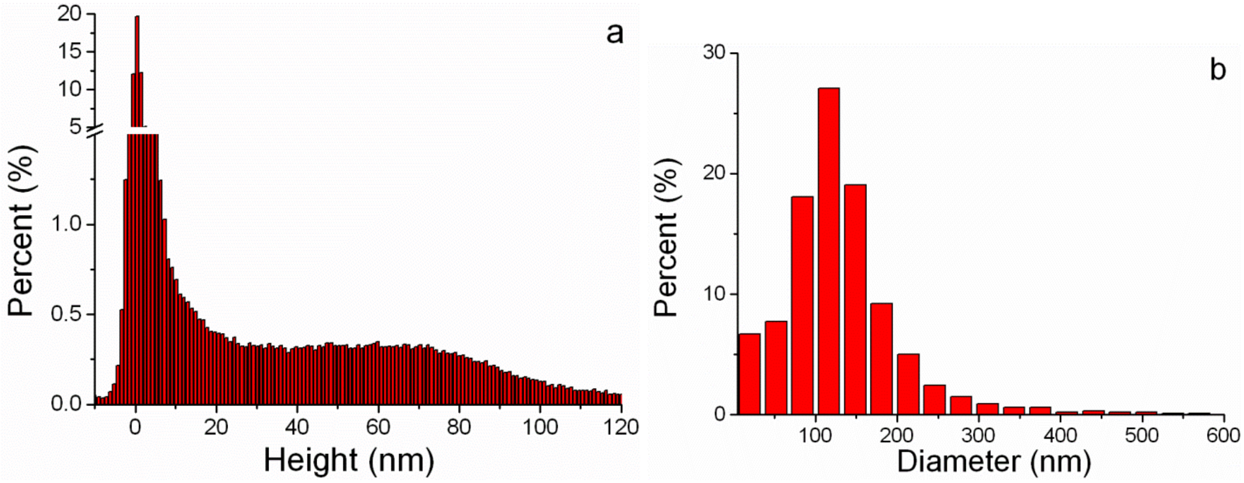

Figure S8. Histograms of the height (**a**) and diameter distribution (**b**) for the sample in Figure 7a in the main manuscript.

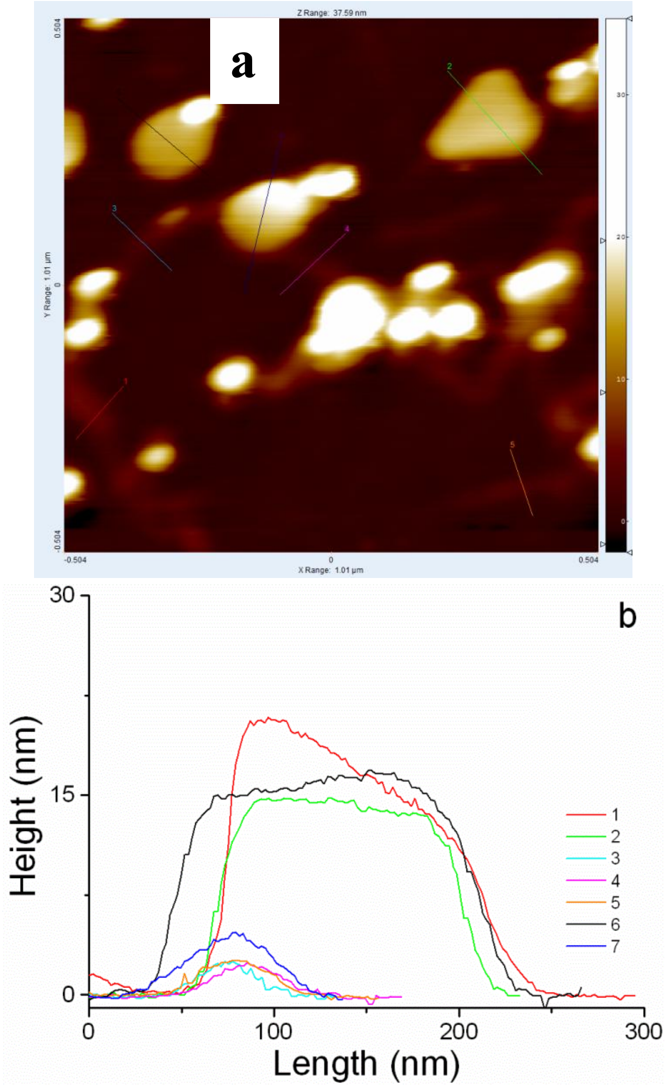

Figure S9. AFM image of the deposits formed on a glass substrate immersed in a Au_{10} and TPPS solution at pH 2.0, showing Au nanostructures and J aggregates marked with colored lines (**a**). Height profiles across the lines shown in the AFM image (**b**).
